# Effects of Zinc Phthalocyanine Photodynamic Therapy on Vital Structures and Processes in Hela Cells

**DOI:** 10.3390/ijms251910650

**Published:** 2024-10-03

**Authors:** Jakub Hosik, Barbora Hosikova, Svatopluk Binder, Rene Lenobel, Marketa Kolarikova, Lukas Malina, Hanna Dilenko, Katerina Langova, Robert Bajgar, Hana Kolarova

**Affiliations:** 1 Department of Medical Biophysics, Faculty of Medicine and Dentistry, Palacky University, 77900 Olomouc, Czech Republic; jakub.hosik@upol.cz (J.H.); svatopluk.binder@upol.cz (S.B.); marketa.kolarikova02@upol.cz (M.K.); lukas.malina@upol.cz (L.M.); hanna.dilenko01@upol.cz (H.D.); katerina.langova@upol.cz (K.L.); bajgar@tunw.upol.cz (R.B.); hana.kolarova@upol.cz (H.K.); 2 Laboratory of Growth Regulators, Faculty of Science, Palacky University and Institute of Experimental Botany of the Czech Academy of Sciences, 77900 Olomouc, Czech Republic; rene.lenobel@upol.cz

**Keywords:** reactive oxygen species, photodynamic therapy, oxidative stress, proteins, mitochondria, DNA, liposome

## Abstract

This work presents results on the efficiency of newly designed zinc phthalocyanine-mediated photodynamic therapy of both tumoral and nontumoral cell models using the MTT assay. Further detailed examinations of mechanistic and cell biological effects were focused on the HELA cervical cancer cell model. Here, ROS production, changes in the mitochondrial membrane potential, the determination of genotoxicity, and protein changes determined by capillary chromatography and tandem mass spectrometry with ESI were analyzed. The results showed that, in vitro, 5 Jcm^−2^ ZnPc PDT caused a significant increase in reactive oxygen species. Still, except for superoxide dismutase, the levels of proteins involved in cell response to oxidative stress did not increase significantly. Furthermore, this therapy damaged mitochondrial membranes, which was proven by a more than 70% voltage-dependent channel protein 1 level decrease and by a 65% mitochondrial membrane potential change 24 h post-therapy. DNA impairment was assessed by an increased level of DNA fragmentation, which might be related to the decreased level of DDB1 (decrease in levels of more than 20% 24 h post-therapy), a protein responsible for maintaining genomic integrity and triggering the DNA repair pathways. Considering these results and the low effective concentration (LC50 = 30 nM), the therapy used is a potentially very promising antitumoral treatment.

## 1. Introduction

Photodynamic therapy (PDT) represents an emerging modality for the treatment of various diseases, including malignancies, neovascular and hyperproliferative disorders, and infections. Compared to conventional therapeutic approaches, PDT offers enhanced selectivity against tumor cells through the utilization of photosensitizers that preferably accumulate in tumor tissues, and the targeted irradiation of tumoral areas with light [1]. The therapy relies on the triple interaction between a nontoxic photosensitizer (PS) or dye, visible light of a specific wavelength absorbed by PS, and molecular oxygen. Following the absorption of light (photons), one of the electrons of the ground state PS is excited into a higher-energy orbital forming the first excited singlet state. This short-lived species (with a lifetime in the nanosecond range) can lose its energy in fluorescence form or by internal conversion into heat. The singlet state PS may also undergo the intersystem crossing process to a more stable (micro- or millisecond) triplet-state with parallel electron spins.

The PS-excited triplet can undergo reactions usually known as type I and type II reactions [2,3,4]. Both types co-occur, and the ratio between these processes depends on the type of PS used and the concentrations of substrate and oxygen. The type I pathway frequently involves the initial production of a superoxide anion by electron transfer from triplet PS to molecular oxygen (monovalent reduction). PS reacts directly with a substrate, such as a cell membrane or a molecule, and transfers a proton or an electron to form a radical anion or radical cation, respectively. These radicals may further react with oxygen to produce reactive oxygen species. Alternatively, in a type II reaction, the triplet PS can transfer its energy directly to molecular oxygen to form excited-state singlet oxygen, and the photosensitizer returns to its ground state. The high reactivity of singlet oxygen leads to interaction with various biological substrates (e.g., proteins, lipids, or DNA) [5,6], causing vital cellular structure impairment and subsequent cell death. The effect of a type II reaction strongly depends on the photosensitizer type, and it is spatially related to the subcellular localization of PS. For instance, PDT with rhodamine derivatives has been shown to lead to NAD(P)H oxidation, but this effect is not observed with Photofrin, Verteporfin, AlPcS4, TPPS4, or protoporphyrin X, despite their mitochondrial accumulation [7]. These findings indicate the importance of the site-specific effects of various photosensitizers [8].

Proteins are primary targets and, hence, quenchers for photo-oxidative reactions, constituting approximately 70% of the dry weight of cells. They often contain endogenous chromophores within the protein structure and exhibit rapid reactions with other oxidized molecules [9,10]. Radical-induced protein modifications can include fragmentation, di- or multimerization, unfolding, structural alternations leading to functional inactivation, changes in mechanical properties, aggregation, cofactor modifications and metal ion binding, formation of other reactive species, or accelerated degradation [11,12]. Reactive oxygen species have the capacity to oxidize cysteine thiols as well as other amino acids like methionine, lysine, arginine, proline, histidine, and tyrosine. For instance, ROS can induce reversible oxidation of cysteine residues in cellular targets such as phosphatases PTEN and PTP1B, kinases like MAPK, and redox-sensitive transcription factors such as FOXO4, thereby influencing various biological processes [13]. ROS are implicated in the modulation of several critical signaling pathways and molecules, including p53, HIF-1α, nuclear factor red 2-related factor 2 (Nrf2)/Kelch-1ike ECh-related protein l (Keap1), nuclear factor-κB (NF-κB), and phosphoinositol 3-kinase (PI3K)/AKT pathways, thus affecting cellular survival and death [14,15]. NF-κB is a crucial transcription factor regulating immune and inflammatory responses. NF-κB proteins are sequestered in the cytoplasm and inhibited by I kappa B (I-κB) proteins. ROS-induced degradation of I-κB results in the translocation of NF-κB to the nucleus, leading to the activation of pro-inflammatory genes and the production of key cytokines such as tumor necrosis factor alpha (TNF-α) and interleukin-1 beta (IL-1β), which can subsequently induce apoptosis [16,17]. Autophagy, a well-known cytoprotective mechanism, helps mitigate intracellular damage and maintain genetic stability under physiological conditions. Several studies have documented the role of ROS in autophagy induction through the activation of the ataxia telangiectasia mutation (ATM) protein, which subsequently activates the TSC2 tumor suppressor via the AMPK metabolic pathway and liver kinase B1 (LKB1). This activation inhibits mammalian rapamycin target protein complex 1 (mTORC1) and triggers autophagy. Additionally, ROS can influence autophagy by regulating Beclin-1, Atg5, and p53. Hydrogen peroxide (H_2_O_2_), through a Beclin-1-dependent mechanism, can induce autophagic cell death, characterized by an increase in the ratio of LC3-II to LC3-I, a decrease in p62 levels, and the formation of autophagic vesicles [14,15,18]. The fragmentation and degradation of proteins mediated by reactive oxygen species are initiated by the abstraction of an α-hydrogen atom from the polypeptide chain. Photodynamic therapy-generated singlet oxygen ^1^O_2_ has been shown to induce oxidative modifications in various proteins across different cellular compartments. The hydroxyl radical (HO•), in conjunction with singlet oxygen (^1^O_2_), has been demonstrated to play a role in the HPD-mediated photodestruction of cytochrome P-450 and related monooxygenases [11]. Additionally, the hydroxyl radical (HO•) appears to be implicated in the induction of mitochondrial inner membrane permeabilization and depolarization, which are processes that occur promptly following the initiation of apoptosis. Superoxide (O_2_^−^) exhibits limited reactivity within biological systems and does not cause significant oxidative damage by itself. The superoxide anion (O_2_^−^) can react with superoxide dismutase (SOD) to generate hydrogen peroxide (H_2_O_2_), thereby influencing the expression of NADPH oxidase (NOX) family proteins and consequently modulating cell apoptosis and proliferation. Superoxide (O_2_^−^) also plays a vital role in generating the highly reactive hydroxyl radical (HO•). In this process, superoxide acts as a reducing agent, not an oxidizing agent. This is achieved by donating an electron to reduce metal ions (such as ferric iron or Fe^3+^) which act as catalysts in the conversion of hydrogen peroxide (H_2_O_2_) into the hydroxyl radical (HO•). This process is known as the Fenton reaction, discovered over a century ago. It is important in biological systems because of iron, copper, or other metals present in most cells, which can catalyze this reaction. The reduced metal (ferrous iron or Fe^2+^) then catalyzes the cleavage of the oxygen–oxygen bond in hydrogen peroxide, resulting in the formation of a hydroxyl radical (HO•) and a hydroxide ion (HO−). Furthermore, the superoxide anion (O_2_^−^) can react with hydroxyl radicals (HO•) to inflict damage on cellular DNA, thereby impairing cellular functions. Excessive production of superoxide anions is associated with aberrant cell apoptosis and has been implicated in the pathogenesis of various diseases [9,18,19]. 

Phthalocyanines, belonging to the second-generation photosensitizers, are particularly promising for PDT due to their robust absorption of tissue-penetrating red light, ease of modification, and high efficiency in generating singlet oxygen [20,21]. Compared to porphyrins, phthalocyanines require lower doses (0.2–0.5 mg/kg), presenting a reduced risk of photosensitivity due to minimal absorption in the 400–600 nm wavelength range [22]. Additionally, their pharmacokinetics are more favorable compared to other photosensitizers like hematoporphyrin derivative (HpD) [23,24].

The effectiveness of photodynamic therapy depends not only on the type and concentration of the photosensitizer used but also on its excitation wavelength (the higher the wavelength, the deeper the penetration in the target tissue) and accessibility of the tumor for irradiation. For this reason, cell lines, tumors located in body cavities, or tumors in which PDT works as an adjunctive treatment following another type of antitumor therapy (e.g., to destroy any residues after surgical removal of the tumor tissue) were chosen. Given that breast and cervical cancers are among the most common female cancers worldwide, there is a need to seek therapy that would increase the recovery ratio. PDT can be used, for example, to treat HPV-related cervical lesions and as a palliative or adjunctive treatment for breast tumors after its surgical resection [25,26]. 

The aim of this study is to determine the suitability of a new photosensitizer derivative for the eradication treatment of cancer cells derived from cervical and breast cancer and analysis of its effect on target cells, when the PS would be considered as potentially suitable for the therapy. Considering that one of the side effects of PDT is the nonselective impact on noncancerous tissue, changes in the viability of two basic types of cells found in the vicinity of tumors, fibroblasts, and keratinocytes were also monitored.

## 2. Results and Discussion

### 2.1. Cell Viability after 5 Jcm^−2^ ZnPc PDT Analysis

**Figure 1:**
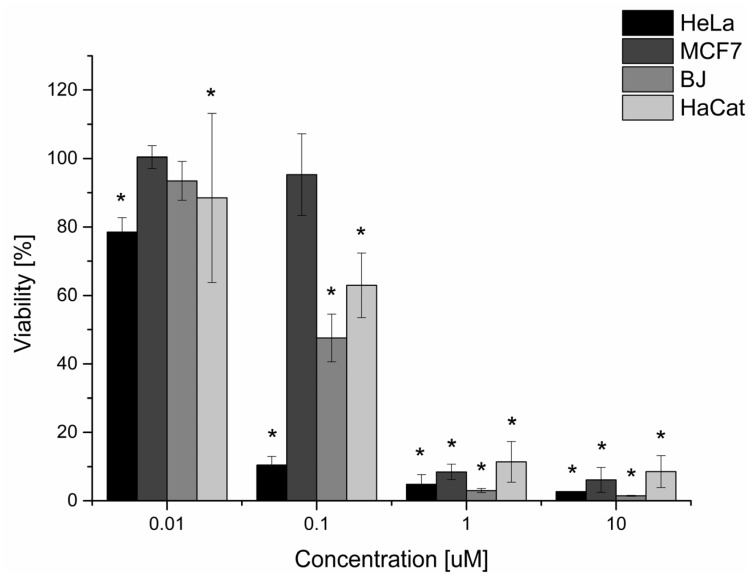
The dependence of tumoral and nontumoral cell viability on the concentrations of the zinc phthalocyanine photosensitizer. The dependence of the cellular viability on the concentration of the zinc phthalocyanine photosensitizer was determined by measuring the enzyme activity of living cells using the MTT test. The irradiation dose used was 5 Jcm^−2^. The control represents the irradiated cells without the photosensitizer (the negative control), and its value is set at 100%. Data are presented as ±SD from three independent measurements. The results were considered statistically significant when *p* < 0.05 and indicated by an asterisk symbol.

The in vitro effect of phthalocyanine-mediated photodynamic therapy (ZnPc PDT) on viability decrease (See Figure 1, Table 1) was investigated on two nontumoral (BJ and HaCat) and two tumoral cell lines (HeLa and MCF7). The reason for including the nontumoral cell lines in the study was the fact that the main disadvantage of photodynamic therapy is the nonspecific uptake of photosensitive substances by nontumoral cell lines, leading to healthy tissue damage. Viability was analyzed through the MTT assay, a colorimetric method, using a certain type of enzymatic reduction that takes place only in living, undamaged cells. Depending on this reduction rate, the color change from yellow to purple occurs (the higher the rate, the more purple). Then, the change in absorbance is assessed. Based on the results of the MTT viability test, we can conclude that the 5 Jcm^−2^ zinc phthalocyanine-mediated photodynamic therapy is the most effective on the HeLa cell line (LC50 = 0.03 µM). On nontumoral cell lines, the LC50 was threefold (BJ) and sixfold (HaCat) higher compared to the HeLa cell line. Based on these results, the zinc phthalocyanine-mediated PDT was considered very prospective for other investigations and cervical cancer treatment. In the case of breast adenocarcinoma cells (MCF7), the LC50 concentration was fifteenfold higher compared to the HeLa cell line. This concentration was also higher compared to the nontumoral cell lines tested. This could lead to massive uptake of the photosensitizer by nontumoral cells when treated by this therapy, hence their extensive damage after the treatment. For this reason, the use of in vitro zinc phthalocyanine-mediated PDT on breast adenocarcinoma cell lines was considered a nonperspective method for other investigations.

### 2.2. HeLa Cell Changes at the Oxidative Stress Level

**Figure 2:**
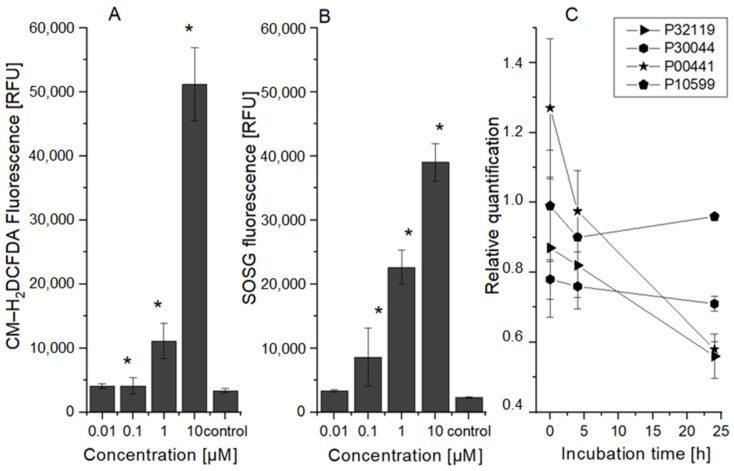
ROS production and protein level changes after 5 Jcm^−2^ ZnPc photodynamic therapy. The dependence of the HeLa cells’ ROS production on the concentration of the zinc phthalocyanine photosensitizer measured immediately after irradiation: (**A**) singlet oxygen production; (**B**) ROS production after type I of PDT reaction. The control represents the irradiated cells without the photosensitizer (the negative control). Data are presented as ±SD from three independent measurements. Results were considered statistically significant when *p* < 0.05 and indicated in the graphs by an asterisk symbol. (**C**) Changes in the proteins involved in oxidation stress reduction depending on incubation time after the therapy. The relative quantification was calculated as the proportion of protein in the control cell sample compared to the protein level in the treated cell sample. P32119 (UniProt identificator)—peroxiredoxin 2, P30044—peroxiredoxin 5, P30041—peroxiredoxin 6, P00441—superoxide dismutase [Cu-Zn], P10599—thioredoxin.

The production of ROS created through type I and type II photodynamic reactions was measured (see Figure 2). Except for the lowest concentration, a significant increase in ROS produced during type I and II reactions was found compared to the control cells. Although singlet oxygen, which is produced by the type II reaction, is generally regarded as the primary agent of photodynamic therapy, the majority of the photosensitive substances caused both types of reactions. Thus, it seems that 5 Jcm^−2^ ZnPc PDT leads to strong production of both reaction types’ ROS products (e.g., H_2_O_2_—a superoxide radical that can react with nitrous oxide in the cell to produce highly reactive peroxynitrite, etc.), depending on PS concentration. This finding is consistent with results from the selected proteins involved in cell response to oxidative stress analysis (see Figure 2). This analysis showed an increase in peroxiredoxin 6 (UniProt identificatory ID P30041) protein, a specific peroxidase responsible for hydrogen peroxide reduction to water and alcohol, 4 h after therapy. It has been found that the antioxidant activity of this protein can play a major role in the repair of lipid peroxidation in the cell membrane and cell survival [27,28,29]. Immediately after therapy, a significant increase in the superoxide dismutase 1 (P00441) protein was found, which efficiently quenches the superoxide radical and converts it to a less toxic hydrogen peroxide. In addition, an increased level of thioredoxin protein (P10599), which plays an important role in reversible S-nitrosylation of cysteine targets, was detected in a sample of cells collected 24 h after therapy. This protein also contributes to NO regulation in cells and thus mediates the reaction of superoxide radicals with NO, leading to strongly reactive peroxynitrite production [30,31,32]. In the case of the other ROS proteins tested, their levels were decreased. These results correspond with Porta et al.’s [33] and Gille et al.’s [34] conclusions and could explain the increased sensitivity of HeLa cells to photodynamic therapy in comparison to the other cell lines used (HaCat, BJ, and MCF7).

### 2.3. HeLa Cell Changes at the Mitochondrial Level

**Figure 3:**
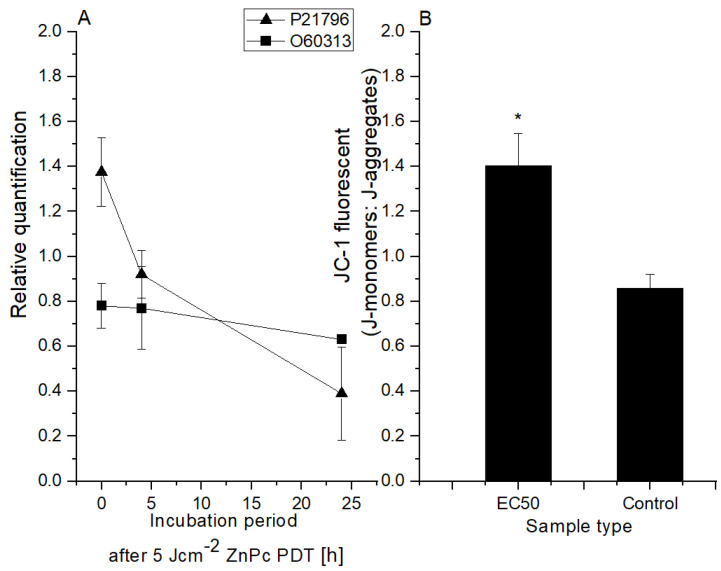
Changes at the mitochondrial level after 5 Jcm^-2^ ZnPc PDT. (**A**) Protein level changes depending on incubation time after the therapy (OPA1 (UniProt ID O60313—dynamin-like 120 kDa protein); VDAC (P21796—voltage-dependent anion-selective channel protein 1)). The relative quantification was calculated as the proportion of protein in the control cell sample compared to the protein level in the treated cell sample. (**B**) The change in HeLa cells’ mitochondria membrane potential using LC50 concentration of zinc phthalocyanine photosensitizer. The control represents the irradiated cells without a photosensitizer (the negative control). Data are presented as ±SD from three independent measurements. Results were considered statistically significant when *p* < 0.05 and indicated in the graphs by an asterisk symbol.

Proton gradient formation across the mitochondrial membrane is an essential energy conservation event that combines carbohydrate and lipid oxidation with ATP production. Preservation and promotion of the membrane potential are critical for mitochondria function. Any change in mitochondrial membrane potential (MMP) caused by any harmful cause, such as an increase in oxygen radicals, can impair mitochondrial function [35,36,37]. In this study, the change in MMP was analyzed through JC-1 fluorescent probe (the higher the JC-1 fluorescence ratio values, the higher the damage to the cells). The results (see Figure 3) showed that compared to the control cells 24 h post-therapy, the JC-1 fluorescent ratio changed by approximately 65% in cells treated with 5 Jcm^−2^ ZnPc PDT at the LC50 concentration. A relationship between mitochondrial dysfunction and proteasome damage has been demonstrated in cell cultures. In particular, the proteasome function is affected by the reduction in mitochondrially dependent ATP syntheses. Mitochondrial failure has been found to be the primary event, followed by proteasome damage [38,39]. These facts are consistent with our results (see Figure 3) when the level of the voltage-dependent anion-selective channel protein 1 VDAC (UniProt identificatory ID P21796) was compared to the control cells immediately after the therapy elevated. However, probably due to mitochondrial membrane damage, a large decrease was found in other samples (4 and 24 h post-therapy). VDAC forms a channel through the mitochondrial outer membrane and the plasma membrane involved in cell volume regulation and apoptosis. This protein plays an important role in induced cell death. Increased VDAC levels could mediate the mitochondrial permeability changes and support mitochondrial-induced cell death by activation of RAS-RAF-MEK pathways. The changes in protein levels responsible for the regulation of mitochondrial permeability may become and serve as an evaluation index of clinical efficacy [19]. In the case of dynamin-related protein OPA1 (UniProt Identification ID O60313, Figure 3), a slight decrease (approx. 20%) was detected immediately after the therapy, but in other time intervals, no further decrease was detected. OPA1 is a protein essential for normal mitochondrial morphology and is required for mitochondrial genome maintenance. Based on these results, in vitro 5 Jcm^−2^ ZnPc PDT at LC50 concentration inflicted damage on the mitochondrial membrane of HeLa cells, leading to disruption of the citrate cycle and ATP production.

### 2.4. Changes in HeLa Cells at the DNA Level

**Figure 4:**
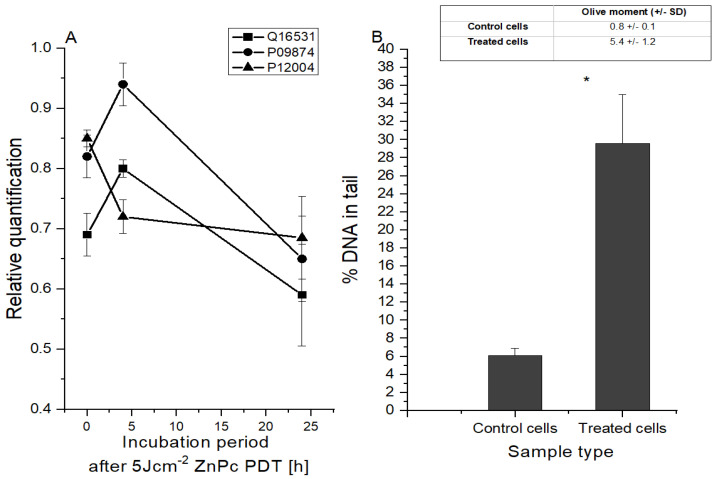
Changes at the DNA level after 5 Jcm^−2^ ZnPc PDT. (**A**) Changes in the protein level depending on the incubation period after the therapy. The relative quantification was calculated as the proportion of protein in the control cell sample compared to the protein level in the treated cell sample. Q16531—DNA damage-binding protein 1; P09874—poly [ADP-ribose] polymerase 1; P12004—proliferating cell nuclear antigen. (**B**) DNA damage evaluated 24 h after therapy by comet assay using LC50 concentration of zinc phthalocyanine photosensitizer. The control represents the irradiated cells without a photosensitizer (the negative control).). Data are presented as ±SD from three independent measurements. Results were considered statistically significant when *p* < 0.05 and indicated in the graphs by an asterisk symbol.

In this study, the effect of in vitro 5 Jcm^−2^ ZnPc PDT at the LC50 concentration on HeLa cell DNA was investigated (see Figure 4). Analysis of selected protein level changes showed that 4 h post-treatment, the levels of proteins that are involved in the detection and triggering of DNA repair pathways (DDB1 and poly [ADP-ribose] polymerase 1) were increased. However, 24 h after therapy, a rapid decrease in both protein levels was detected. The level of proliferative nuclear antigen protein, which plays a crucial role in the response to DNA damage through coordination of replication with DNA repair and DNA damage tolerance pathways, has gradually decreased. DDB1 protein, which binds to DDB2 to form the UV-damaged DNA-binding protein complex, is important for the maintenance of genomic integrity, and it was found that DDB1 protein defective cells accumulate double-stranded breaks [40,41]. This correlates with the findings from the comet analysis, which allows for the detection of DNA fragmentation rates. It was found that 24 h after therapy, the amount of fragmented DNA increased from 6.1 to 29.6% compared to in the control cells.

### 2.5. Cellular Localization and Molecular Function of Degraded Proteins

#### 2.5.1. Cellular Localization

**Figure 5:**
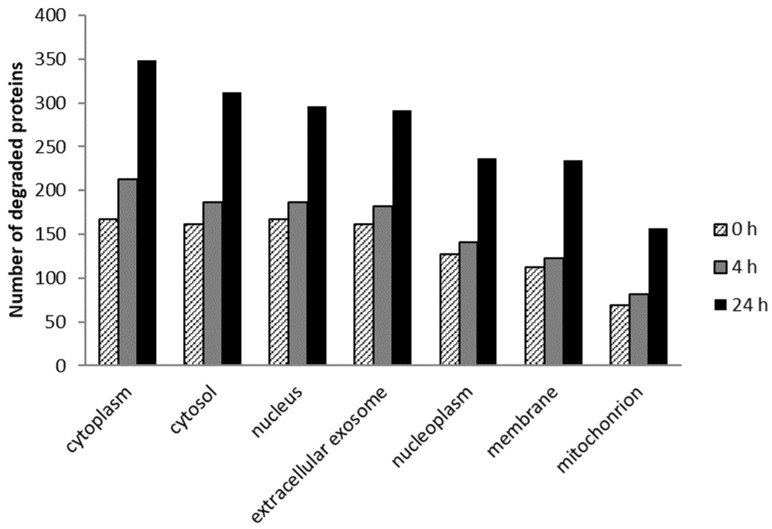
The seven largest groups of the degraded proteins in terms of cell localization (G0 terms) identified in samples with 0, 4, and 24 h incubation time after in vitro 5 Jcm^−2^ ZnPc PDT at the LC50 concentration on HeLa cells. Degraded proteins were identified by comparing the measured sample to the control cells without photosensitizer and irradiation. The proteins identified were sorted using David Bioinformatic Resources 6.8 when *p* < 0.0001 [44,45].

In this research, the cellular localization of HeLa cell degraded proteins was analyzed at three incubation intervals (0, 4, and 24 h) after 5 Jcm^−2^ ZnPc PDT at the LC50 concentration (see Figure 5). The results showed that the most degraded proteins with cellular localization were found in the cytoplasm, cytosol, nucleus, extracellular exosome, nucleoplasm, membranes, and mitochondria in all samples. The results are consistent with the findings that phthalocyanine photosensitizers are not usually accumulated in only one cell organelle but affect multiple cellular structures [42]. At 4 h after the therapy, we observed an increase (compared to 0 h post-therapy) in the number of degraded proteins, ranging from 9% of membrane proteins to 27% of cytoplasmatic proteins. Twenty-four hours after the treatment, the number of degraded proteins was substantially higher—ranging from 75% of nuclear proteins to 128% of mitochondrial proteins. This is consistent with the fact that cell reactions to impairment, such as triggering the reparative mechanisms of apoptosis, usually take place over longer time periods (e.g., in the case of apoptosis, 6–24 h, depending on the cell type) [43].

#### 2.5.2. Molecular Function

**Figure 6:**
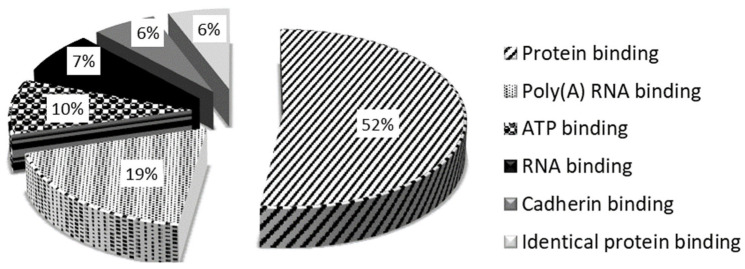
The six largest groups of the degraded proteins in terms of molecular function (G0 terms) identified 24 h after in vitro 5 Jcm^−2^ ZnPc PDT at the LC50 concentration on HeLa cells. Degraded proteins were identified by comparing the measured sample to the control cells without photosensitizer and irradiation and sorted using David Bioinformatic Resources 6.8 when *p* < 0.0001 [44,45].

The molecular function of degraded proteins was studied 24 h after 5 Jcm^−2^ ZnPc PDT at the LC50 concentration on HeLa cells (in Figure 6, the six largest groups are shown). In the cell sample collected 24 h after therapy, the absence of the proteins involved in identical protein binding and ATP binding, which serve as essential coenzymes and enzyme regulators (NAD kinases, DEADbox helicases, adenosine kinase (ADK), etc.), was observed. These findings are consistent with the results of mitochondrial membrane damage analysis, where 4 h after the therapy, the disruption of the citrate cycle and ATP production was observed. ATP and NAD(P)H molecules are essential activated carriers in the cell that are involved in many biosynthetic reactions, and their damage leads to the impairment of the cellular anabolic–catabolic system and, thereby, to the disruption of vital cell processes with subsequent cell death [46,47]. The identified proteins were sorted using David Bioinformatic Resources 6.8 when *p* ˂ 0.0001 [44,45].

### 2.6. Incorporation of the Sensitizer into the Liposomes

**Figure 7:**
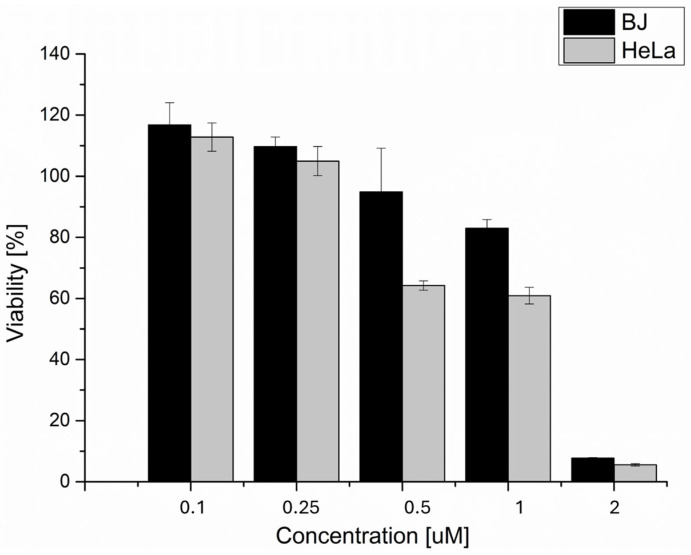
The dependence of tumoral HeLa and nontumoral BJ cells’ viability on the concentrations of the zinc phthalocyanine photosensitizer after sensitizer incorporation into the liposomes. The cellular viability was determined by measuring the enzyme activity of living cells using the MTT test. The irradiation dose used was 5 Jcm^−2^. The liposomal content was released from liposomes using ultrasound (3 Wcm^−2^, 60 s, pulse mode). The control represents the irradiated and sonicated cells without the photosensitizer (the negative control). Data are presented as ±SD from three independent measurements.

The incorporation effect of ZnPc into DPPC liposomes on cellular viability after the therapy was studied. Considering that the DPPC transition temperature (T_t_ = 41.3 °C) is above the body and cultivating temperature, and this temperature would lead to cell death by itself, the liposomal content should be released from the liposomes differently than heating above the T_t_. In this study, high-frequency ultrasound (1 MHz, 3 Wcm^−2^) supported by 2H,3H-perfluoropentane (PFC5) addition was applied to release the sensitizer from the liposomes. Although the photodynamic therapy alone was very effective, the LC50 value increased after the incorporation into the liposomes (see Figure 7). Nevertheless, one of the primary functions of the drug incorporation into the liposomes is to protect the incorporated drug against the reaction with other molecules and rapid drug clearance from the bloodstream [48]. Although the LC50 was higher than in the case of the only drug application, its encapsulation into the DPPC liposomes could be very beneficial [49]. Encapsulation into the liposomes enables drug protection against degradation, site-targeting, and also, with the application of ultrasound, the controlled release in target tissue [50,51]. The lethal concentration of the ZnPc sensitizer on the noncancerous BJ cell line was closer (LC50 = 1.5839 uM) but still more than 50% higher than on the cancerous line HeLa (LC50 = 1.04 uM). Considering the fact that the targeting of cervical cancer cells could be easily achieved by the ultrasound, the liposomal ZnPc therapy could be prospectively used as cervical cancer therapy. Moreover, for the drug release, the ultrasound device BTL4000 was used. This device could be commonly found in most hospitals, and it is not expensive to acquire. This fact makes the therapy applicable in almost every medical care center.

## 3. Materials and Methods

### 3.1. Photosensitive Substance and Cell Lines

**Figure 8:**
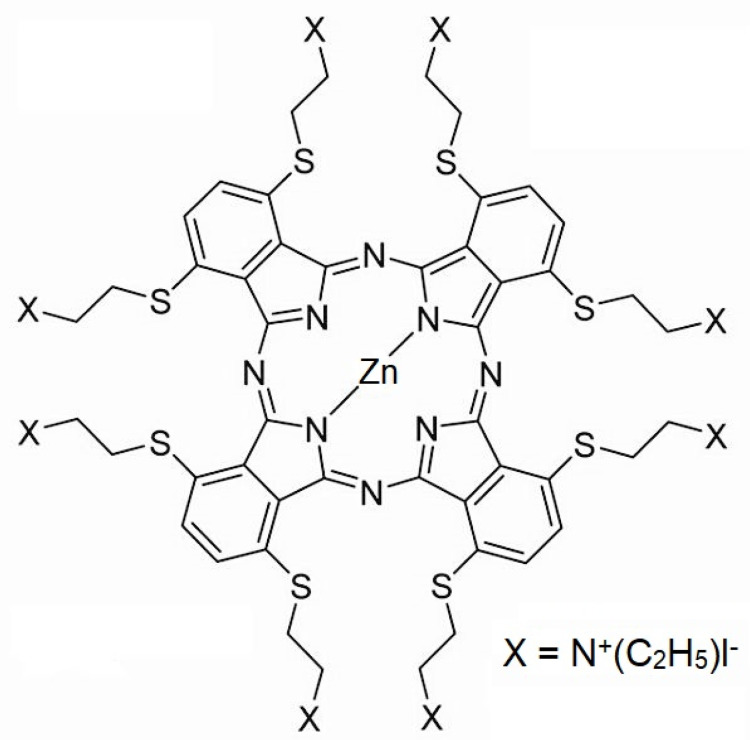
The chemical structure of the zinc phthalocyanine (ZnPc) used in the study. The chemical formula of ZnPc: 2,3,9,10,16,17,23,24-Octakis[(2-(triethylammonio)ethyl)sulfanyl]phthalocyaninato]zinc(II) Octaiodide [52].

The new phthalocyanine zinc photosensitizer (ZnPc) was synthesized by A. Cidlina [52]. This photosensitizer does not aggregate in water due to eight substituents in nonperipheral positions. Before use, the photosensitizer was diluted in 1X phosphate-buffered saline (PBS). The chemical structure and formula of the phthalocyanine used are presented in Figure 8.

For the initial in vitro viability analysis, the four cell lines were used. HeLa (cervical cancer), BJ (fibroblasts), and MCF7 (breast cancer) were purchased from the American Type Culture Collection (ATCC, Manassas, VA, USA). HaCat cells (keratinocytes) were purchased from Cytion (CLS, Eppelheim, Germany). These cell lines were cultivated in Dulbecco’s modified Eagle’s medium (DMEM) containing photosensitizer for 24 h in darkness at 37 °C and 5% CO_2_.

### 3.2. Light Source and Exposure

An LED-based light device designed specifically for irradiation of experimental microplates was used (device protected by National Patent No. CZ 302829 B6). The samples were illuminated at 660 nm wavelength. The cells with photosensitizer were exposed to a total irradiation dose of 5 Jcm^−2^ (irradiation time 334 s, irradiation intensity 15 mWcm^−2^) at room temperature.

### 3.3. MTT Viability Test

The phototoxic effect of the photosensitizer used on the tumoral cell lines HeLa (cervical cancer cells; ATCC, Manassas, VA, USA) and MCF7 (breast adenocarcinoma; ATCC, Manassas, VA, USA) and nontumoral BJ (human skin fibroblasts; ATCC, Manassas, VA, USA) and HaCat (epidermal keratinocytes; Cytion, Germany) cell lines was measured by MTT assay (Sigma Aldrich, Burlington, MA, USA). Cells were cultivated in 96-well microplates (1 × 10^4^ cells/well). For the MTT test, the decadic logarithmic concentration scale was used. Twenty-four hours after 5 Jcm^−2^ ZnPc PDT, the DMEM (Sigma Aldrich, Burlington, MA, USA) was replaced by a solution containing 50 µL of 0.5 mgml^−1^ MTT dissolved in 1X PBS. The cell lines with MTT were incubated for 4 h at 5% CO_2_ and 37 °C. The MTT solution was then replaced with 100 µL of DMSO to help dissolve the formazan crystals. The absorbance level (570 nm) was measured by a Tecan Infinite Pro200 (Tecan, Männedorf, Switzerland). The LC50 (the photosensitizer concentration killing half of the cell population at 5 Jcm^−2^) values were calculated by Phototox 2.0 software (BfR, Berlin, Germany) from the dose–response dependence curve.

### 3.4. ROS Measurement

#### 3.4.1. Type I Reaction Products’ Measurement

The HeLa cells were incubated with the photosensitizer in darkness at 37 °C and 5% CO_2_ in 96-well microplates (1 × 10^4^ cells/well). After 24 h, the cultivating medium (DMEM) was replaced by a solution with a 10 µM fluorescence probe CM–H_2_DCFDA (Ex/Em: ~495/530 nm; Invitrogen, Waltham, MA, USA) in 1X PBS. The cells were incubated with the probe solution for 30 min and irradiated. Immediately after the irradiation, the ROS production level was measured by the microplate reader Tecan Infinite Pro200 (measurement parameters: fluorescence top reading mode; excitation bandwidth 9 nm; emission bandwidth 20 nm, integration time 20 µs; Z-position (manual) 20,000 µm).

#### 3.4.2. Type II Reaction Product (Singlet Oxygen) Measurement

As the CM–H_2_DCFDA probe is not able to detect the singlet oxygen, the SOSG probe (Singlet Oxygen Sensor Green, Ex/Em: ~500/525 nm; Life Technologies, Carlsbad, CA, USA) was used for its detection. The cells were incubated with the photosensitizer in darkness at 37 °C and 5% CO_2_ in 96-well microplates (1 × 10^4^ cells/well). After 24 h, the cultivating medium (DMEM) was replaced by a solution with a 10 µM fluorescence probe SOSG dissolved in 1X PBS. The cells were incubated with the probe solution for a further 20 min and irradiated. Immediately after the irradiation, the fluorescence level was measured using the microplate reader Tecan Infinite Pro200 (measurement parameters: fluorescence top reading mode; excitation bandwidth 9 nm; emission bandwidth 20 nm, integration time 20 µs; Z-position (manual) 20,000 µm).

### 3.5. Comet Assay

The DNA damage caused to the tumor and nontumor cells was evaluated using a comet assay. The HeLa cells were incubated with the photosensitizer in darkness at 37 °C and 5% CO_2_ in 96-well microplates (1 × 10^4^ cells/well) for 24 h. The DMEM was then replaced by 1X PBS, and the cells were irradiated by means of LEDs with a maximum emission of 660 nm. The irradiation dose was set at 5 Jcm^−2^. Following the irradiation, 1X PBS was replaced by the DMEM, and the cell lines were incubated for 24 h.

The microscope slides were first precoated with 1% HMP (high-melting-point) agarose (SERVA Electrophoresis, Heidelberg, Germany) dissolved in distilled water and placed in a drying oven for 30 min at 60 °C. A total of 85 µL of 1% HMP agarose in 1X PBS was applied on the precoated slides, covered with a cover slip, and placed in a refrigerator to encourage agarose gelling. The cells were trypled (TrypLe, Gibco, Thermofisher, Waltham, MA, USA) to detach the cell from the bottom of the well. The effect of TrypLe (Thermofisher, Waltham, MA, USA) was stopped using DMEM containing a fetal bovine serum (FBS). The isolated cells were centrifuged for 4 min at 1500 rpm. The cell pellet was then dispersed in 20 µL of 1X PBS and vortexed. A total of 85 µL of 1% LMP (low-melting-point) agarose (Sigma Aldrich, Burlington, MA, USA) was added to this solution, and 85 µL of the suspension was placed on the solidified agarose on the microscope slide and covered by a cover slip. The microscope slides were then transferred to a refrigerator for 15 min. The samples without the cover slips were, after solidifying, immersed in a lysis buffer (2.5 M NaCl (Sigma Aldrich, Burlington, MA, USA), 100 mM EDTA (ethylenediaminetetraacetic acid) (Sigma Aldrich, Burlington, MA, USA), 10 mM Tris (tris (hydroxymethyl) aminomethane) (Sigma Aldrich, Burlington, MA, USA), and 1% Triton X-100 (SERVA Electrophoresis, Heidelberg, Germany), pH 10) at 4 °C for a period of 60 min. After the lysis, the slides were placed in an electrophoretic tank and dipped for 40 min in a cold electrophoretic solution (300 mM NaOH (Sigma Aldrich, Burlington, MA, USA) and 1 mM EDTA (Sigma Aldrich, Burlington, MA, USA)). The electrophoresis was run at 350 mA and 0.8 Vcm^−1^ for 20 min. Following completion of the electrophoretic separation, the slides were carefully rinsed twice for 10 min with a neutralization buffer (0.4 M Tris, pH 7.5; Sigma Aldrich, Burlington, MA, USA) at 4 °C. The samples were stained using fluorescent probe SYBR Green (Sigma Aldrich, Burlington, MA, USA) and visualized using a fluorescence microscope with a CCD camera (Olympus, Japan). Cells were manually scored using CometScore 1.5 software (TriTek, Dayton, Ohio, USA). Median values of the Olive moment, the amount of the DNA in the tail, which is directly proportional to the DNA damage, were evaluated.

### 3.6. Protein Analysis

For the protein analysis, the HeLa cells were incubated with photosensitizer at the LC50 concentration (30 nM) in darkness at 37 °C and 5% CO_2_ in a Petri dish (1 × 10^7^ cells/well) for 24 h and then irradiated. Subsequently, the cells were collected at 3 different times—immediately after the irradiation, 4 h after the irradiation, and 24 h after the irradiation. The cells were centrifuged for 2 min at 1500 rpm. Subsequently, the supernatant was removed, and pellets were stored at −80 °C. The protein concentration was measured through a 2-D Quant Kit (Invitrogen TM, Waltham, MA, USA). The extraction and purification methods were identical to those described by Petřík [53]. The proteins were labeled according to Boersema et al.’s [54] protocol. Samples were measured by capillary chromatography and tandem mass spectrometry with ESI (UHR-QTOF maXis, Bruker Daltonics, Bremen, Germany) on an analytical column filled with RP (reversed phase; C18). Data were collected using the DDA (data-dependent acquisition) method with cyclic MS (mass spectrometry) collection and a variable number of MSMS (tandem MS) specters in the 2 s cycle. Primary data were processed by DataAnalysis (Bruker Daltonics, Bremen, Germany) and extracted from MGF (files containing a list of precursors and their fragmentation spectra). Using ProteinScape v3.1, MASCOT (MatrixScience, London, UK) was identified using the HUMAN reference database obtained from the UniProt repository. Protein quantification was performed using a Bruker tool set, and the quantitative data were processed using the Perseus v. 1.3.5 software (Max Planck Institute of Biochemistry, Planegg, Germany). The relative quantification was calculated as the proportion of protein in the treated cell sample compared to the protein level in the control cell sample. Protein analysis was performed using DAVID 6.8 Functional Annotation Bioinformatics Microarray [44,45]. The sets of all proteins determined after each time period after the therapy were uploaded to this microarray and compared with the protein set found in control cells. Lost proteins from the treated sample were then sorted by their molecular function and cellular localization.

### 3.7. Mitochondrial Membrane Potential Measurement

Mitochondrial membrane potential was evaluated using fluorescence probe JC-1 (5,5´,6,6´- tetrachloro-1,1´,3,3´ tetraethylbenzimidazolylcarbocyanine chloride; Biotium, Fremont, CA, USA). JC-1 exists as a monomer at low concentrations and yields green fluorescence (emission at 530 nm), similar to fluorescein. At higher concentrations or higher mitochondrial potential, JC-1 forms J-aggregates that exhibit a broad excitation spectrum and an emission maximum at ~590 nm. HeLa cells were incubated in 96-well microplates (1 × 10^4^ cells/well) for 24 h with ZnPc and then irradiated. Immediately after irradiation, DMEM was replaced with 1X PBS medium with JC-1 at the final assay concentration of 2 µg/mL for 20 min at 37 °C and 5% CO_2_, and then washed with 1X PBS, and red and green fluorescence was measured through Tecan Infinite reader. Results were expressed as the ratio of green to red fluorescence (530/590 nm).

### 3.8. Incorporation of the Sensitizer into the Liposomes

For the liposomal therapy, 1,2-dipalmitoyl-sn-glycero-3-phosphatidylcholine (DPPC) lipids at a concentration of 10 mg/mL were used (Avanti Polar Lipids, Alabaster, AL, USA). Firstly, 2H,3H-perfluoropentane (PFC5) was inserted into the core liposome. The size of the core liposomes with PFC5 was determined by the extrusion method (Avanti, Alabaster, AL, USA). PFC5 serves as an ultrasound agent enhancing the ultrasound effect [51]. Then, these 100 nm PFC5 liposomes were mixed with sensitizer (1 mM) and DPPC lipids. The liposomes were then created by the extrusion method to the final size of 200 nm. The ZnPc–liposome mixture was diluted in 96% ethanol to achieve the sensitizer concentration for liposome determination. The sample fluorescence was then measured using a Tecan Infinite Pro200 reader. The ZnPc liposomal concentration was compared with the known concentration of the unbound photosensitizer diluted in 96% ethanol to confirm proper concentration determination. The HeLa cells were then incubated with ZnPc liposomes in 96-well microplates (1 × 10^4^ cells/well) for 24 h in darkness at 37 °C and 5% CO_2_. After 24 h, the DMEM was replaced by 1X PBS. The cells were exposed to high-frequency ultrasound (1 min, 3 Wcm^−2^, pulse mode) and irradiation (660 nm, 5 Jcm^−2^). The ultrasound intensity and frequency setup were chosen based on the previous experiments, where the liposomal content was released without damaging cells.

### 3.9. Statistical Analysis

The data were analyzed using the statistical software IBM SPSS Statistics for Windows, Version 23.0. (IBM Corp, Armonk, NY, USA). The differences between two independent sets of quantitative quantities were verified by a two-sample *t*-test. Differences between multiple independent sets were searched using ANOVA. Subsequently, Dunnett´s post hoc test was performed to compare every sample to the control. Statistically significant results (*p* ˂ 0.05) are indicated in the graphs using an asterisk symbol.

## 4. Conclusions

An in vitro antitumoral treatment with a new zinc phthalocyanine derivative, which was synthesized to increase its water solubility, led to a reduction in protein levels invoked in defensive response to oxidative stress (e.g., rapid decrease in superoxide dismutase four hours after the therapy). Furthermore, in these cells, the proteins involved in the maintenance of mitochondrial integrity and membrane potential were gradually degraded after the therapy. A significant reduction in proliferative nuclear antigen protein (over 20% lower level 24 h post-therapy) was found to be related to an increase in DNA breaks in cervical cancer cells.

Currently used antitumoral therapies (e.g., radiotherapy, chemotherapy, currently approved PDT, etc.) usually have many side effects. Among these, affection of non-neoplastic tissues, degradation of photosensitizer to toxic metabolites, or secondary metabolism of the photosensitizer associated with unwanted reactions leading to the formation of other molecules before reaching the target site were described to hinder positive therapeutic outcomes. Photodynamic therapy, the effects of which were evaluated in this in vitro study, is effective at a low concentration of the drug (LC50 = 30 nM), affects more cervical cancer cells than noncancer cells (threefold higher LC50 in fibroblasts and sixfold in keratinocytes), damages the basic cellular processes of cancer cells, and enables simple incorporation of the used drug into a protective biodegradable liposomal carrier. In addition, the combination with ultrasound-guided therapy could enhance the treatment as ultrasound penetrates deeper into soft tissues thereby reaching hardly accessible sites. Considering the above-mentioned results, the evaluated therapy appears to be potentially very promising for the treatment of cervical cancer.

## 5. Patents

The liposomes used in the study are protected by a National Utility patent.

## Figures and Tables

**Table 1:** LC50 values for tumoral and nontumoral cell lines treated by 5 Jcm^−2^ ZnPc. Data are presented as mean values ±SD from three independent measurements.

Cell Line	LC50 [µM] ZnPc
BJ	0.09 ± 0.02
HaCat	0.17 ± 0.03
MCF7	0.45 ± 0.17
HeLa	0.03 ± 0.01

## Data Availability

Research data are available from the corresponding author on request.
